# Exploring biorepository donation patterns, experiences, and recommendations: a mixed-methods study among Appalachian adults enrolled in a sugary drink reduction program

**DOI:** 10.3389/fpubh.2024.1371768

**Published:** 2024-05-09

**Authors:** Donna-Jean P. Brock, Theresa Markwalter, Li Li, Samyukta Venkatesh, Cheyanne Helms, Annie Reid, Jamie M. Zoellner

**Affiliations:** ^1^School of Medicine, Public Health Sciences, University of Virginia, Charlottesville, VA, United States; ^2^School of Medicine, Family Medicine, University of Virginia, Charlottesville, VA, United States

**Keywords:** biomarker research, biospecimens, engagement, rural, medically underserved areas

## Abstract

**Background:**

Under-represented subgroups in biomarker research linked to behavioral health trials may impact the promise of precision health. This mixed methods study examines biorepository donations across an Appalachian sample enrolled in a sugary drink reduction intervention trial.

**Methods:**

Participants enrolled in the behavioral trial were asked to join an optional biomarker study and were tracked for enrollment and biospecimen returns (stool and/or buccal sample). At 6 months, participants completed a summative interview on decision-making process, experiences collecting samples, and recommendations to encourage biospecimen donation. Return rates were analyzed across demographics (i.e., age, gender, race, education, income, health literacy status, and rurality status) using chi-squares. Qualitative data were content coded with differences compared by biomarker study enrollment and donation choices.

**Results:**

Of the 249 invited participants, 171 (61%) enrolled, and 63% (*n* = 157) returned buccal samples and 49% (*n* = 122) returned stool samples. Metro residing participants were significantly more likely (56%) to return stool samples compared to non-metro (39%) counterparts [*x^2^*_(1)_ = 6.61; *p* = 0.01]. Buccal sample return had a similar trend, 67 and 57%, respectively for metro vs. non-metro [*x^2^*_(1)_ = 2.84; *p* = 0.09]. An additional trend indicated that older (≥40 years) participants were more likely (55%) to donate stool samples than younger (43%) participants [*x^2^*_(1)_ = 3.39; *p* = 0.07]. No other demographics were significantly associated with biospecimen return. Qualitative data indicated that societal (66–81%) and personal (41–51%) benefits were the most reported reasons for deciding to donate one or both samples, whereas mistrust (3–11%) and negative perceptions of the collection process (44–71%) were cited the most by those who declined one or both samples. Clear instructions (60%) and simple collection kits (73%) were donation facilitators while challenges included difficult stool collection kits (16%) and inconveniently located FedEx centers (16%). Recommendations to encourage future biorepository donation were to clarify benefits to science and others (58%), provide commensurate incentives (25%), explain purpose (19%) and privacy protections (20%), and assure ease in sample collection (19%).

**Conclusion:**

Study findings suggest the need for biomarker research awareness campaigns. Researchers planning for future biomarker studies in medically underserved regions, like Appalachia, may be able to apply findings to optimize enrollment.

## 1 Introduction

Using a precision health approach, the application of biological data to verify and tailor behavioral health interventions has the potential to reduce morbidity and mortality, particularly among high-risk populations (e.g., racial and ethnic minorities, rural communities, uninsured or underinsured individuals, and those with lower educational attainment, health literacy, and socio-economic status) (1–3). As such, the promise of precision health is greater when biological data are integrated with individual, social, and environmental contexts with the purpose of reducing health disparities (1, 3). Unfortunately, clinical studies, including biomarker research, suffers from inequities in recruitment from subgroups that are low health literate, low income, racial and ethnic minorities, and living in medically underserved and/or rural areas. This lack of representation prevents these subgroups from accessing precision health benefits and puts them at risk for persistent health disparities (2, 4–9).

One subgroup of particular concern is individuals residing in medically underserved and/or rural regions. Medically underserved areas have a shortage of primary health services and face economic, cultural, or language barriers to health care (e.g., low income) (10). Over 80% of rural counties in the United States are designated as medically underserved (10). Not only are these areas plagued by healthcare deserts, they also face additional barriers such as geographic distances and lack of transportation as well as low socio-economic status and educational attainment that result in lower rates of preventive care and cancer screening along with higher rates of chronic illness and premature mortality (7, 8). However, despite the urgent need for greater representation of these communities in biomarker research, there are few studies examining the barriers and motivators of rural participants for donating biological samples (11).

Studies attempting to understand the willingness of medically underserved and/or rural populations to donate biospecimens for research have relied primarily on focus group and survey data (4, 6–8, 11). These studies have generally found a willingness to participate in biomarker research that is fueled by altruism, yet tempered by a lack of knowledge, geographical distance from research centers, and mistrust in the research process. While these are important insights, findings are generated from participants hypothetical willingness to enroll in biomarker research as a means of predicting motivations for actual enrollment. Generalizing this willingness data into actual behaviors is further complicated by biospecimen requests that are unspecified or narrow in variety (7, 8, 11). The current study aims to address this gap in the literature by examining attitudes, motivations, and expectations toward biomarker research across biorepository donation decisions among medically underserved Appalachian participants, including many who reside in rural areas.

This biomarker research takes place within the context of enrollment in iSIPsmarter, a clinical behavioral randomized control trial (RCT) focused on reducing sugar sweetened beverage (SSB) intake for Appalachian adults in Southwest Virginia and Eastern West Virginia. While this Appalachian area has diverse counties that range from highly rural to micropolitan in nature, much of the region is medically underserved. As a subgroup, the targeted Appalachian region has SSB intake twice as high as national estimates (12), high prevalence of and mortality from numerous SSB-related chronic conditions, and bears disproportionate burden from compromised determinants of health [e.g., high poverty rates (13), low educational attainment (14), and low health literacy (15)]. While specifics of the RCT are described elsewhere (16), participants enrolled in the behavioral intervention were asked to participate in an optional biomarker study by donating either a stool sample, buccal sample, or both across three different time points. The goal of this biorepository is to develop a representative Appalachian sample of high SSB consumers for which impacts on gut microbiota and epigenetics can be explored and in relationship to behavior changes. This study tracked baseline biological donations and conducted telephone interviews with all participants enrolled in iSIPsmarter to obtain feedback on their decision to donate to the biorepository, their experience collecting samples, and their feelings about future participation in biomarker research.

The primary objectives of this mixed-methods paper were to (1) quantitatively examine biomarker study enrollment among a rural Appalachian population, (2) determine if there are demographic or rurality variances for stool and buccal donations, and (3) qualitatively examine participants’ described reasons for whether to donate to the biorepository. Secondary objectives included qualitative analyses of participants’ descriptions of the sample collection process and recommendations on how to improve engagement in biomarker research. Other researchers planning for future biomarker studies in rural and medically underserved populations may be able to apply findings to optimize enrollment and proactively address subject barriers.

## 2 Materials and methods

### 2.1 Study design

This mixed methods exploratory evaluation used quantitative sample return tracking and survey data to determine biorepository enrollment and donation rates as well as potential demographic disparities. All participants who were enrolled in the larger iSIPsmarter trial were given the option to enroll in the biomarker study. Biomarker study participants were asked to donate their biospecimens in conjunction with iSIPsmarter baseline, 6-month, and 18-month data collection. This study focuses on biospecimen donations and demographics collected at baseline. Additional qualitative interview data were collected from all iSIPsmarter participants, regardless of whether they enrolled in the optional study, at the 6 month follow-up. Interviews were analyzed for context in baseline donation decisions and processes, as well as potential means for improving biomarker research participation among a rural Appalachian sample. The study protocol was approved by the University of Virginia Institutional Review Board. Participants provided written informed consent and received a $25 gift card for each sample donated to the biorepository.

### 2.2 Study participants

**Figure 1:**
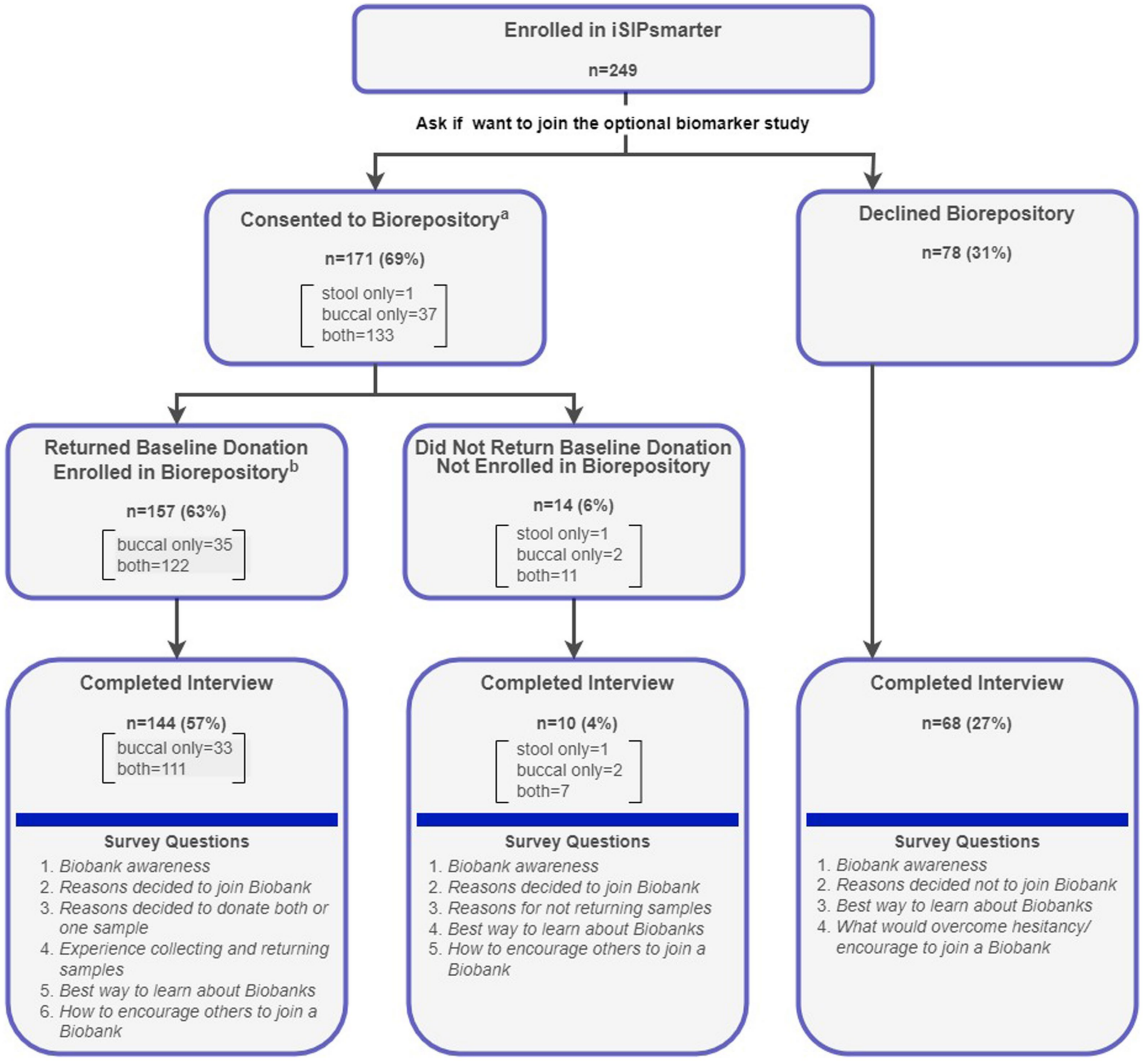
Consort diagram for biorepository enrollment, baseline sample return, and interview completion with question flow. ^a^During the consent process, participants could choose to donate a stool and/or a buccal sample. ^b^Enrollment into the Biobank study was not complete until a baseline sample was returned.

The current biorepository study sample was recruited from the 249 participants enrolled in the parent iSIPsmarter study (see Figure 1). The biorepository study was presented as an optional component to the iSIPsmarter trial. Enrolled iSIPsmarter participants were recruited through research team led efforts such as social media and press releases (15%), word of mouth (8%), and community partner promotions (77%). Over one half (59%) of enrolled participants were referred through higher education (35%) and health care community partners (24%). These included promotions through community college and university health and wellness initiatives as well as referrals from Federally Qualified Health Centers and regional Departments of Health. The remaining 19% of community partner referred participants came from public schools and early education (9%), local government agencies (5%), and non-profit or local businesses (4%).

### 2.3 Biorepository enrollment, sample collection, and data collection processes

As seen from the consort diagram (Figure 1), the iSIPsmarter participants who were invited to join the biomarker study could choose to donate a stool sample, buccal sample, or both. Participants who consented to donate to the biorepository were mailed collection kit(s) via FedEx with instructions for collecting and returning the samples. Samples were self-collected and returned to the laboratory using a pre-paid FedEx envelope. Participants were considered fully enrolled in the biomarker study once they returned their baseline sample(s). A total of 222 (89%) of the 249 iSIPsmarter participants completed the qualitative interview, including 144 who were enrolled and 78 who were not enrolled in the biorepository study (see Figure 1). All qualitative data were collected alongside iSIPsmarter 6-month follow-up assessment. Baseline demographic data were collected as part of the iSIPsmarter electronic surveys.

### 2.4 Measures

Quantitative data were collected from surveys and tracking documents for the 249 enrolled iSIPsmarter participants. Demographic data were collected during an online enrollment screening for iSIPsmarter as well as the baseline iSIPsmarter survey. Demographics included race, gender, age, education level, income, and rurality. Rurality was operationalized using the 2023 Rural–Urban Continuum Codes (RUCC), which rank counties by population size and degree of urbanization and adjacency to metro areas (17). Based on participant’s home address, RUCCs were assigned to the participants and categorized as metro (RUCC 1–3) or non-metro (RUCC 4–9) (17). Associations with health literacy levels were also collected using baseline scores on the Newest Vital Sign (NVS), a six-item questionnaire based on the Nutrition Facts Label. Scores from the NVS were categorized into established cut-offs of low (0–3 correct) or high (4–6 correct) health literacy (18). Biorepository enrollment and sample donation were tracked by research coordinators for all iSIPsmarter participants.

Participant perception and experience with the biomarker research was assessed through a semi-structured interview. This interview was included as part of the 6-month follow-up assessment. An interview guide was developed by three (one masters level and two doctoral level) study team members (see Appendix A). The interview guide assessed information across three domains: participants’ decision-making processes related to enrollment in the biomarker study, facilitators and barriers to sample collection, and proposed strategies to support enrollment and reduce hesitancy in joining a biomarker study. Domains were based on prior research examining willingness to donate biospecimens (8, 11, 19, 20). Additional questions regarding how to best communicate about biomarker research were also included. The interview was formatted to be straightforward, brief, and with few prompts. Questions were tailored to the participants’ experience (i.e., participants who did not complete the biobank were not asked about facilitators and barriers to collection). These decisions were made to increase the efficiency of the interviews and to eliminate the need for audio recording. Importantly, the interview was part of a longer non-audio recorded assessment call and the team felt it would be prohibitive to ask to record only this section. The team had successfully collected qualitative data previously during summative evaluations without recording (21).

Research staff, four Master-level [Master of Public Health (MPH), MS] and one doctoral-level (DSc), conducted the semi-structured telephone interview with participants. Interviewers took structured field notes during the interviews and attempted to capture direct quotes of key replies. To support the reliability of data, interviewers were trained on best practices for interviewing procedures, including how to use the interview guide and field note process.

### 2.5 Analyses

Quantitative data were analyzed using Chi-squares to determine associations between donation returns (buccal sample and stool sample) and demographics, including rurality status. *A priori p*-values of <0.05 were interpreted as statistically significant. Additionally, given the somewhat limited sample size and exploratory nature of this study, and in efforts to avoid Type 2 error, *p* values <0.10 were presented as trending (22).

Hand written field notes were entered verbatim into an excel spreadsheet. Two Master-level researchers reviewed the data and developed an initial codebook based on emerging content categories within the three domains outlined by the semi-structured interview (Table 1). To support the trustworthiness of the coding, researchers coded in pairs. Using the codebook, responses were manually content coded independently. Differences in codes were identified, discussed together, and rectified for agreement by the researchers. As needed, the codebook was revised. Revisions included collapsing codes into larger categories based upon code frequencies and overlap in meaning (23). For example, Trust-Negative under Domain 1 was developed from two codes: (1) mistrust in researchers ability to protect participant privacy and (2) perceptions that biomarker research is unregulated (see Table 1 for original and final codes). As seen in Table 1, prior to rectification, interrater reliability of the final codes were generally very high. Using McHugh’s more conservative interpretation of the kappa statistic, 20 of the 23 codes had “strong” (*k* = 0.80–0.90; *n* = 11) to “moderate” (*k* = 0.60–0.79; *n* = 9) interrater reliability, while the remaining three were “weak” (*k* = 0.40–0.59; *n* = 1) or “minimal” (*k* = 0.21–0.39; *n* = 2) (24). Final rectified coding of the data was uploaded into SPSS 29 for descriptive analysis across enrollment and donation choices.

**Table 1:** Formation of final interview domain codebooks and inter-rater reliability prior to rectification.

Original codebook	Final codebook	Kappa
**Domain 1: Decision making process codes**		
Helpful to science/others	Societal benefit-positive	0.83
Not helpful to science/others	Societal benefit-negative	N/A^a^
Personal/family medical reason Incentives Personal interest	Personal benefit-positive	0.82
No personal benefit Not interested	Personal benefit-negative	0.85
Comfortable with collection Perceived ease of collection	Collection process-positive	0.82
Uncomfortable with collection Perceived difficulty of collection Complicating health issue Takes too much time Hassel to return	Collection process-negative	0.84
Trust researchers/process Understanding of protections	Trust-positive	0.72
Mistrust researchers/privacy concerns Biomarker research perceived as unregulated	Trust-negative	0.87
**Domain 2: Facilitators and barriers to sample collection**		
Collection kits that were simple, easy to use, and intuitive	Collection kits that were simple, easy to use, and intuitive	0.68
Clear instructions provided through writing, images, and video	Clear instructions provided through writing, images, and video	0.77
Easy and accessible sample return system through FedEx	Easy and accessible sample return system through FedEx	0.61
Collection experience was unpleasant	Collection experience was unpleasant	0.90
Faulty or difficult to use collection kits Collection kits were confusing and not intuitive	Collection kits were challenging to use or had malfunctioning parts	0.60
Difficult and inaccessible sample return system through FedEx	Difficult and inaccessible sample return system through FedEx	0.88
Instructions were overwhelming Did not understand instructions	Instructions were unclear or confusing	0.60
**Domain 3: Strategies to support and reduce hesitancy in joining biomarker research**		
Emphasize biobank benefits to science/others Provide evidence of benefits of biobanking	Examples of Biobank benefits for others	0.83
Improved monetary incentives	Improved monetary incentives	0.88
Provision of sample test results	Provision of sample test results	0.74
Make collection process easy and quick Emphasize simplicity of collection process	Simple collection processes	0.23
Have strong privacy protections Explain regulations that protect biospecimens	Ensured privacy and security of samples	0.80
Clear purpose for sample collection	Clear purpose for sample collection	0.59
Upfront specifics on use of samples Provide updates on sample use	Upfront and ongoing clarity on sample use	0.72
Simplify consent language Ensure understanding of consent	Simplified consent language	0.31
Testimonials from other participants	Testimonials from other participants	0.71

^a^No participants refuted the societal benefits of biomarker research within the interviews.

## 3 Results

### 3.1 Biomarker study participation and demographic variability across stool and buccal donations

The majority of enrolled participants were female (83%), White (89%), and non-Hispanic (96%) with a mean age of 42 years (SD = 12.60). Over one half (59%) were designated as residing in metro or more urban counties with the remaining 41% designated as residing in non-metro or more rural counties. Most also had more than a high school education (91%) with 59% having a 4-year college degree. Newest Vital Sign (NVS) scores also indicate high levels of health literacy (88%). Although demographics on household size were not collected, given an average of a two person household, reported annual household income indicates that approximately half of our sample would be living below the federal poverty line (9%; < $20,000) or be considered low-income (41%; <$55,000; < 300% of federal poverty line).

As seen in Figure 1, 171 (69%) of the 249 participants consented to donate to the biomarker study. Of these, the majority (*n* = 133; 78%) agreed to donate both samples. A total of 157 (63%) of the iSIPsmarter participants returned either a buccal sample (*n* = 35) or both a buccal and stool sample (*n* = 122) at baseline and were considered fully enrolled. In total, 157 (63%) buccal samples and 122 (49%) stool samples were donated.

**Figure 2:**
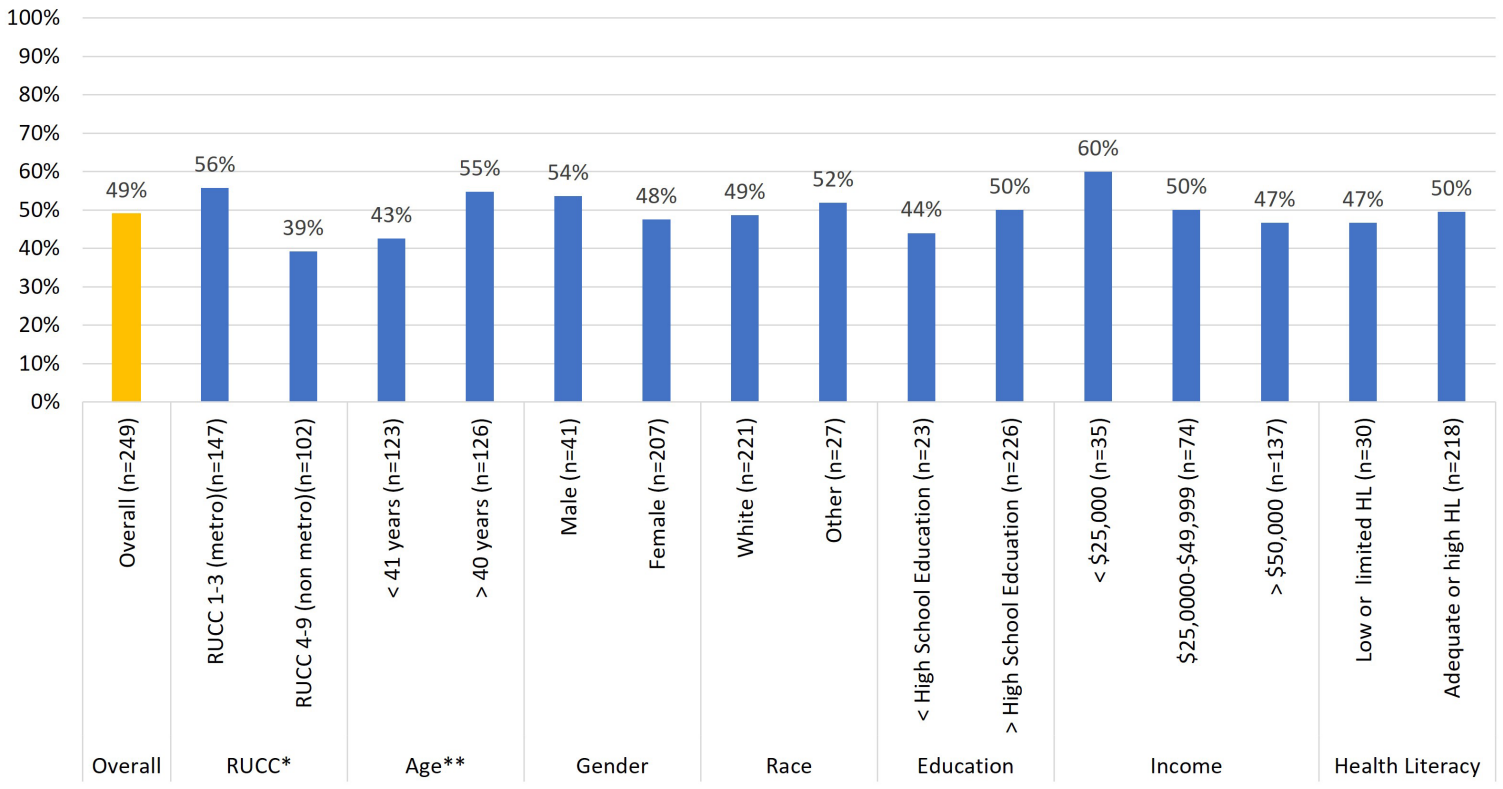
Relationships between baseline stool sample donation and participant demographic characteristics (*n* = 249). ^*^p < 0.05, ^**^p < 0.10.

**Figure 3:**
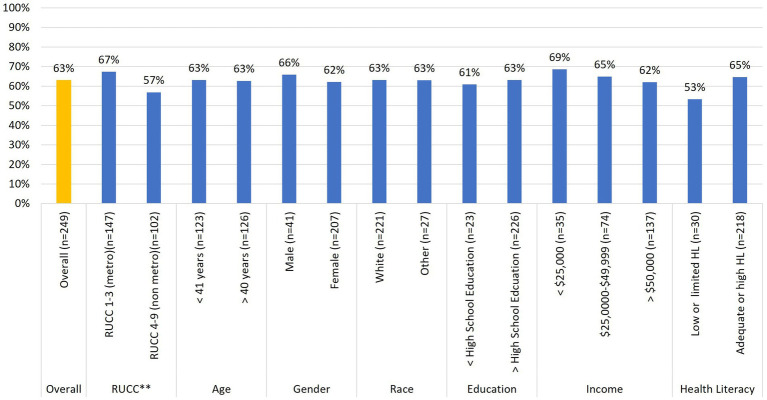
Relationships between baseline buccal sample donation and participant demographic characteristics (*n* = 249). ^*^p < 0.05, ^**^p < 0.10.

Figures 2, 3 summarize differences in demographics across those who returned either stool samples (Figure 2) or buccal samples (Figure 3). Regarding stool sample returns (Figure 2), Chi-square analyses revealed no statistically significant associations across baseline demographics for age, gender, race, education level, family household income, and health literacy. However, there was one statistically significant [*x*^2^_(1)_ = 6.61; *p* = 0.01] association between rurality status and stool sample returns. iSIPsmarter participants living in metro designated counties were more likely (56%) to return these samples than were their non-metro counterparts (39%). In addition to rurality status, there was a trend [*x*^2^_(1)_ = 3.39; *p* = 0.07] indicating that older participants (greater than 40 years) were more likely (55%) to donate a stool sample than were younger participants (43%) (less than 40 years).

Regarding buccal sample donations (Figure 3), Chi-square analyses revealed no statistically significant associations across any of the demographic variables. However, while not statistically significant, there was a trend [*x*^2^_(1)_ = 2.84; *p* = 0.09] similar to that between stool sample donation and rurality status. iSIPsmarter participants living in metro designated counties were more likely (67%) to return a buccal sample than those living in non-metro designated counties (57%).

### 3.2 Awareness of biomarker research

Those who completed the summative interview during the 6-month follow-up assessment (*n* = 222) were asked to respond yes or no as to whether they had prior knowledge of biomarker research. The vast majority (86%) of these participants indicated that they were unaware of such research. Chi-square analyses for associations with baseline demographic characteristics revealed a trend [*x^2^*_(1)_ = 3.25; *p* = 0.07] suggesting that those who had prior knowledge of biomarker research were more likely to be from metro (74%) as opposed to non-metro (26%) counties. Additionally, those who had prior knowledge of biomarker research were also more likely (81%) to donate samples than those who did not (62%) [*x^2^*_(1)_ = 3.94; *p* = 0.02]. No other demographics were significantly associated with prior knowledge of biomarker research.

### 3.3 Decision factors for donating to the biorepository

**Figure 4:**
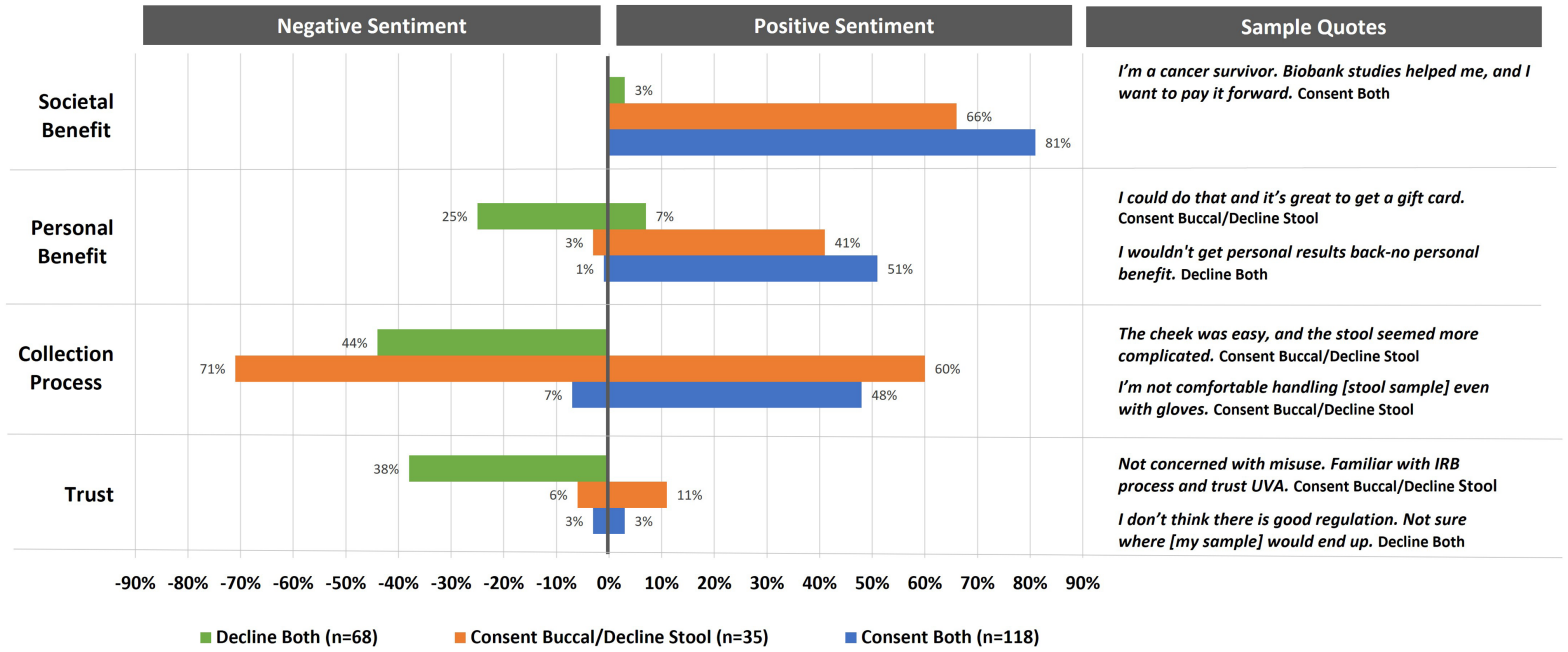
Percent of interviewed participants indicating positive or negative sentiments toward enrolling in biomarker research (*n* = 221). Annotation under the title a stool sample/declined a buccal sample. Due to the small sample size, they are not included in analysis.

As seen from Figure 4, participant descriptions of their decision-making processes for whether to donate to the biorepository were sorted into four categories: (1) societal benefits (the desire to advance science and help others), (2) personal benefits (monetary incentives, intellectual curiosity, and test results), (3) perceived collection process (ease and comfort), and (4) trust in the use and handling of samples. These categories were coded as having a positive sentiment if benefits for donating were identified, collection processes were perceived as easy, or trust in biomarker research was mentioned. In contrast, categories were coded as having negative sentiment if benefits for donating were not perceived or were not in balance with risks or challenges, collection processes were perceived as difficult, and there was stated mistrust in biorepository practices.

Sentiment coding on biorepository decision-making categories varied across participants who declined donation (*n* = 68), agreed to donate one sample (*n* = 35), or agreed to donate both samples (*n* = 118) (Figure 4). Overall, societal benefit was the most mentioned reason for deciding to donate. Positive sentiments were stronger for those who decided to donate both buccal and stool samples (81%) as opposed to those who just agreed to donate a buccal sample (66%). Upon further examination, almost one-third (30%) of these altruistic participants (*n* = 119) indicated that their desire to help stemmed from a health diagnosis for themselves or someone close to them (16%) or an affiliation with a medical or research field (14%). While 3% of those who declined to donate samples indicated that biomarker research has societal benefits, none of the participants, regardless of donation choices, indicated negative sentiments for this category. Similar to societal benefits, participants in all biorepository donation groups acknowledged personal benefits to biomarker research (both = 51%, buccal only = 41%, decline both = 7%); however, one quarter of those who declined to donate indicated that the personal benefits were lacking with 3% of those who decided to donate one sample and 1% of those who decided to donate both samples indicating the same.

Positive perceptions about the collection process were mentioned most frequently (60%) by those who chose to donate just a buccal sample (see Figure 4). This same group of participants also had the most negative perceptions of the collection process regarding the stool sample (71%). While 48% of those who chose to donate both samples felt positively about the collection process, only 7% had reservations. In contrast, 44% of those who decided to decline both samples had negative perceptions of the collection process, with none expressing any expectations that sample donation would be simple or straight forward.

Finally, trust in biomarker research was infrequently mentioned as a reason for deciding to donate one (11%) or both (3%) samples (see Figure 4). However, mistrust was mentioned by 38% of those who declined participation. An additional 6% of those who chose to donate just the buccal sample and 3% of those who chose to donate both samples also indicated reservations with how biospecimens were used and secured.

### 3.4 Collection barriers and facilitators

The 144 participants who returned a baseline sample to the biorepository and completed the 6-month interview were asked to report on any facilitators or barriers to the collection process. As seen in Table 2, the facilitators mentioned most often included collection kits that were simple, easy to use, and/or intuitive (73%) and clear instructions provided through writing, images, and video (60%). An additional 14% also indicated that they appreciated the ease of being able to return the samples through FedEx via a pick-up call, drop boxes, or distribution center. Consistent with participants’ perceptions of greater difficulty with the stool sample collection as compared to the buccal sample (Figure 4), 83% of those that reported the importance of clear instructions were stool sample donators while 85% of the buccal only donators indicated that the ease of the collection was a facilitator (Table 1).

**Table 2:** Facilitators and barriers to the sample collection experience for participants who donated both samples (*n* = 111) or only the buccal sample (*n* = 33) to the biorepository.

Theme description		Total	Sample donation		Sample quotes
		*(n = 144)*	*Both*	*Buccal only*	
Facilitators	Collection kits that were simple, easy to use, and intuitive.	73%	69%	85%	*No big deal, pretty self-explanatory*. Both *Collection tools were easy to use.* Both
	Clear instructions provided through writing, images, and video.	60%	83%	17%	*Video was helpful. Instructions were clear-nice visuals.* Buccal Only
	Easy and accessible sample return system through FedEx.	14%	14%	15%	*Super easy, well explained. Using FedEx for pick-up was also really easy and convenient.* Both
Barriers	Collection kits were challenging to use or had malfunctioning parts.	16%	20%	3%	*The toilet accessory did not want to “cooperate.” It did not stick to the toilet seat [and it] fell into the toilet.* Both
	Difficult and inaccessible sample return system through FedEx.	16%	11%	33%	*It took patience and understanding for mailing back kits. I had to go to two different FedExs.* Both
	Sample collection was unpleasant.	8%	11%	0%	*All the instructions were good. It was gross, but I did it.* Both
	Instructions were unclear or confusing.	5%	5%	3%	*Need to emphasize the amount of stool needed, provide example and more instructions.* Both

Barriers to the collection process were much less reported than facilitators (Table 2). As seen in Table 1, only 16% indicated that the collection kits were challenging, with an additional 16% noting challenges with returning samples. An additional 8% reported the unpleasantness of the collection as a barrier while 5% found instructions unclear or confusing. Further exploration of barriers revealed that similar to the facilitators to collection, those who experienced collection difficulties (95%) and unpleasant experiences (100%) were primarily from participants who collected a stool sample (i.e., both samples). Of those that reported collection difficulties, almost one half (43%) were due to malfunctions with the toilet accessory component of the collection kit that is used to capture the stool while an additional 22% were confused about the amount of stool sample to provide. Of those that reported difficulties returning their samples, this was primarily due to FedEx drop-off centers that would not accept overnight packages or distribution centers that were geographically distant. An examination across rurality status for this barrier did not reveal differences between metro (14%) vs. non-metro (19%) participants that would indicate this barrier was more concentrated in rural areas.

### 3.5 Future biorepository donation: encouraging others and overcoming hesitancies

As seen in Table 3, most biorepository donors mentioned that messages around benefits, such as how biomarker research helps science and others (58%), would help encourage participation. One quarter (25%) of these same participants also indicated that higher value incentives would spark more interest. Those who were not enrolled also believed that these factors could potentially encourage others and help them overcome their hesitancy, albeit to a lesser extent (benefits to others 33%; monetary incentives 12%). Both those enrolled and not enrolled were similar in their endorsement of easy collection processes and trust building factors such as sample security, clear purpose, and transparency about sample use (Table 2). Differences were noted for simplicity in consent language. Those who were not enrolled in the biomarker study were more likely to mention the importance of this (14%) than those who were enrolled (6%). Though mentioned to a lesser extent, a small number of participants indicated that testimonials from others and being able to see results from sample testing would be helpful in deciding whether to donate to a research biorepository.

**Table 3:** Ways to encourage or overcome hesitancy for biorepository participation as mentioned by enrolled (*n* = 144) and not enrolled (*n* = 78) participants.

Theme description		Total	Enrollment status		Sample quotes
		*(n = 222)*	*Enrolled*	*Not enrolled*	
Benefits	Examples of Biobank benefits for others.	50%	58%	33%	*Sharing benefits of the research/study and how it can help people and their communities.* Not enrolled
	Improved monetary incentives.	20%	25%	12%	*Higher value incentive for stool sample donation.* Enrolled
	Provision of sample test results.	3%	2%	5%	*Getting feedback on biobank testing would really change my mind about participating. Has a more win-win feeling about it.* Not enrolled
Collection	Simple collection processes.	19%	22%	15%	*Make sure to say how easy stool sample is to collect.* Enrolled
					*Explain process is not gross, easy to take sample.* Not enrolled
Trust	Ensured privacy and security of samples.	20%	17%	24%	*Maybe helping us understand how it is being used. What kind of research. Who is using it and how it is disposed of. I think people worry about these things--not everyone has same “good” intentions.* Not enrolled
	Clear purpose for sample collection.	19%	17%	23%	*If there was a future check in about what your samples were being used for instead of a general statement with no plan.* Not enrolled
	Upfront and ongoing clarity on sample use.	14%	13%	17%	*Find a way to explain the purpose in common terms.* Not enrolled
	Simplified consent language.	9%	6%	14%	*Testimonials about how easy it was.* Enrolled
	Testimonials from other participants.	4%	3%	5%	

Participants indicated that the best ways to hear about biomarker studies were through a combination of promotions using social media (38%); emails (35%); print, radio, and television advertisements (19%); phone calls/texts (11%); live outreach events (7%); and mailed materials (6%). Almost one third (31%) emphasized an academic-community partnership in which trusted local stakeholders such as employers and health care providers (24%) and friends or family members (7%) would explain and promote biospecimen donation. Almost one quarter (24%) of participants who did not enroll in the biomarker study (*n* = 78) indicated that more education about biorepositories is necessary to raise awareness of the importance of this research and encourage participants to donate.

## 4 Discussion

Overall, our study’s findings indicated that almost two-thirds (63%) of the targeted Appalachian iSIPsmarter participants donated at least one sample to the optional biomarker study. Additionally, participants were more likely to return a buccal sample (63%) compared to a stool sample (49%). Our findings of actual biospecimen donations are somewhat lower than a previous study in which 73% of surveyed Appalachian adults hypothetically indicated that they would be willing to donate samples to a biorepository (11). Moreover, in contrast to this previous study that found no difference in participant’s hypothetical willingness to donate types of samples (i.e., blood, saliva, or buccal) (10), our study found participants were less likely to donate a stool sample relative to a buccal sample. Importantly, our findings move beyond hypothetical willingness and into actual biospecimen donation. Also, our study’s examination of qualitative data on donation decision-making and collection experiences expands on previous findings. For example, not only were our participants less likely to donate a stool sample, but they also perceived and experienced this type of donation as more burdensome than the buccal sample. Examination of participant suggestions to improve engagement in future biomarker research includes use of localized awareness campaigns; transparency and security around sample use and storage; easy to use collection kits and procedures; and trusted community recruitment and research partners.

Despite the moderately high participation rates in our study, differences in biospecimen donations existed between metro and non-metro participants. In general, metro residing participants were more likely to donate stool and buccal samples than were their non-metro counterparts. This is consistent with the literature concerning the underrepresentation of rural samples in Biobank research (5–8). In addition to the disparity in rurality, there was a trend indicating that younger (less than 40 years old) participants were less likely to donate a stool sample than older participants. Other literature on the relationship between age and biospecimen donation are mixed (11, 25–28). For instance, age differences were not noted for the hypothetical willingness of an Appalachian sample to donate blood, saliva, or buccal samples (11). In contrast, other studies indicate that persons aged 40–65 are more favorable toward biospecimen donation, though these studies are not specific to stool sample donations (25, 26). In studies specific to stool bank donation for fecal microbiota transplantation, no age differences were noted for willingness to donate a stool sample for targeted participants in Canada, England, United States, and Australia (27, 28). These inconsistent findings related to age may indicate more complex relationships between participant characteristics and donation of biological samples. Future studies should consider multivariate analyses that include demographic, contextual, and attitudinal factors associated with biospecimen donation.

In addition to the disparities in donations, our qualitative data indicated a general lack of knowledge about biomarker research among participants. This finding was more salient for non-metro participants than for their metro counterparts. Moreover, those who were not aware of biomarker research were less likely to donate a sample. Literature reviews suggest that deficiencies in basic understanding of research related biorepositories is prevalent across Europe and the United States, with up to two-thirds of Americans lacking basic knowledge (25). In rural areas where research infrastructure is limited or geographically distant, it is not surprising to find disparities in biorepository awareness. This lack of knowledge seems to be a primary factor for insufficient biorepository diversity and may outrank unwillingness or mistrust of under-represented populations to donate biospecimens (6, 29).

Our qualitative feedback data from participants about their decision-making processes and experiences provide context to the quantitative findings. In sum, our participants are highly motivated to donate samples for altruistic reasons. While those who did not donate samples did not deny the societal benefits of biomarker research, they admitted to being unknowledgeable as to the specifics of those benefits. As such, those who decided not to engage in donations were most influenced by mistrust of the process and perceptions that sample collection would be difficult or uncomfortable. Many of these findings are similar to what is found in biomarker studies on donation willingness (4, 8, 11, 19, 25, 26). Our participants’ suggestions for improving biorepository engagement provide insight into the motivations of our medically-underserved sample and have led to the following recommendations:

1. Improve awareness of biomarker research through educational campaigns. To counter the lack of awareness in biomarker research, educational campaigns specific to the context of targeted communities may be necessary. As seen from the emerging themes of our qualitative data, educational messages should address both benefits and procedures of biorepositories in ways that are easy to understand. The Boot Camp Translation project provides a Community Based Participatory Research Approach (CBPR) model for translating the complex medical language of biorepositories into culturally tailored evidence-based communications (30). Using this approach can tap into key concerns of a community, such as privacy, while encouraging participation for the greater good of society. It can also provide helpful information about the best approaches for message format and tone. When these campaigns are specific to a biorepository donation request of the population, collection procedures can also be explained to manage perceptions and expectations about the collection process.
2. Develop consent procedures that are transparent about biorepository purpose and sample use and security. Clinical trial consent protocols, including those for biorepositories, are often the result of institutional templates that are consistent with federal guidelines, but not entirely responsive to individual studies or the needs of targeted populations (31). Participants are demanding more transparency and agency in donation of biospecimens (8, 25, 32, 33). To overcome hesitancies around mistrust, our participants indicate a need for simplified language in consenting that is transparent about privacy, purpose, and use of samples. Participant aspirations for transparency in biomarker research are shared by researchers who seek basic standards in biorepository consenting that would ensure understanding of purpose, risks, and benefits (31). Unfortunately, research has shown that even after a simplified consent process, participants were not able to demonstrate adequate comprehension of these standards (34). A dynamic consent approach that breaks down the consent process over time and provides participants with continued agency over their sample use has the potential to improve transparency of biomarker research and produce greater levels of trust among underrepresented populations (35).
3. Ensure that collection kits are reliable and easy to use, and instructions are clear. Our participants who donated samples overwhelmingly indicated that clear, multi-modality instructions as well as simple straight forward collection kits were key to successful sample donation. Some of our participants’ negative experiences with the collection kits were unavoidable. However, researchers’ knowledge of the collection kits prior to sending them to participants may help to identify potential kit malfunctions and collection errors and find solutions to prevent unusable samples and participant frustrations. Difficulties with FedEx delivery and return also highlighted the importance of knowing the targeted communities and identifying alternative mailing solutions that are accessible to participants.
4. Leverage community partners to recruit participants: this suggestion is in alignment with our participant recommendations to utilize community stakeholders to engage participants in biomarker research. Misgivings about collection of biological samples may be ameliorated when the request comes from trusted community organizations. Studies on disparities in clinical trials support the need for community level intermediaries, such as Federally Qualified Health Centers, faith based organizations, and community service agencies for increasing representation of medically underserved populations (6). The recruitment strategies and enrollment success of our biomarker study were consistent with this literature.

While our findings and suggestions may be helpful for future biomarker researchers, several limitations should be considered when interpreting our study findings. First, our sample is specific to Appalachia and is not racially or ethnically diverse. Findings may not be generalizable outside of the targeted region. Similarly, the combination of recruitment partners (e.g., higher education) and participants interested in the parent iSIPsmarter study skewed toward female and college educated individuals who may not be representative of the region’s general population. Second, our definitions of rurality were based on RUCC codes that rely on county population estimates. Using singular definitions of rurality within the boundaries of county lines can result in skewed rurality estimates that may be exacerbated by our choice to collapse RUCC codes into metro and non-metro categories for ease of comparisons (36). Third, this exploratory study was not specifically powered to detect differences in stool and buccal sample biorepository donations, thus the null and trending quantitative findings should be cautiously interpreted. Fourth, the decision not to audio record the interviews may have impacted specificity or context around emerging themes. Finally, qualitative data are limited to participants’ experience and are not directly related to the quantitative analysis for disparities. Specifically, qualitative data questions were not designed to capture positive and negative attitudes about biorepository participation. While sentiments around reasons for donating were coded, these are spontaneous, and thus opposing sentiments (e.g., negative for those who donated, positive for those who declined), are most likely under-represented. Similarly, the quantification of the qualitative data may provide insight into communalities in thinking among our participants, but cannot suggest the importance of one category, challenge, or suggestion over another. These limitations should be interpreted within the strengths of this study including concentration on a medically underserved Appalachian population, use of mixed methods to examine actual donation for two different types of biological samples, and adequate representation of qualitative perspectives among those who declined sample donation.

In sum, findings from this study bring attention to potential continued rural disparities in biorepository representation. Participant feedback suggests the need to find approaches to educate and build trust around biomarker research within health disparate communities. This study provides unique information about decision-making processes and experiences from participants in a medically underserved Appalachian region who were asked to join a biomarker study within the context of a larger behavioral science clinical trial. Biorepository representation from this vulnerable under-served populations is important to understand how social determinants of health may impact disease process and biological mechanisms. When paired with behavior trials, it has the potential to identify evidence-based precision health interventions. Participants’ reflections and suggestions are key to addressing barriers for participating in biomarker research and understanding ways to improve biorepository engagement.

## Data availability statement

The raw data supporting the conclusions of this article will be made available by the authors, without undue reservation.

## Ethics statement

The studies involving humans were approved by Institutional Review Board for Health Sciences Research-University of Virginia. The studies were conducted in accordance with the local legislation and institutional requirements. The participants provided their written informed consent to participate in this study.

## Author contributions

D-JB: Data curation, Formal analysis, Investigation, Methodology, Project administration, Visualization, Writing – original draft, Writing – review & editing. TM: Data curation, Formal analysis, Investigation, Methodology, Visualization, Writing – original draft, Writing – review & editing. LL: Conceptualization, Funding acquisition, Investigation, Methodology, Resources, Supervision, Writing – review & editing. SV: Data curation, Project administration, Writing – review & editing. CH: Formal analysis, Writing – review & editing. AR: Data curation, Project administration, Writing – review & editing. JZ: Conceptualization, Funding acquisition, Investigation, Methodology, Resources, Supervision, Writing – original draft, Writing – review & editing.
