# Supplementary material for: Exploring biorepository donation patterns, experiences, and recommendations: a mixed-methods study among Appalachian adults enrolled in a sugary drink reduction program
Source: Front Public Health. 2024 May 9;12:1371768. doi: 10.3389/fpubh.2024.1371768 (PMC11111869; doi:10.3389/fpubh.2024.1371768)
Supplement: Supplementary file 1 [file Table_1.DOCX]

**Appendix A: Interview Guide for Enrolled and Declined Biobank Participants**

**Biobank 6-month Follow-up Summative Interview for Enrolled Participants**

Recruitment PID__________ Enrollment PID_______________ Date: _________

Samples Agreed to Donate: CHEEK STOOL

Samples Received by Biobank at Baseline: CHEEK STOOL

Recollection Request: YES NO Recollection Received: YES NO

**INTRO AND PURPOSE STATEMENT:**

As part of the consent process for joining this study, you learned about an Additional optional study you could participate in with the purpose of creating a Biobank for future research. A Biobank is like a regular bank, but instead of depositing money for future spending, we are depositing biological samples for future research. The general idea of this Biobank was to collect gut microbiome and genetic samples through stool sample and cheek swab sample donations. This would potentially allow researchers to study the effects of sugary drinks on gut health and genetic functioning and would allow researchers to follow iSIPsmarter participants to examine how lifestyle changes may impact overall health.

This is our lab's first experience with enrolling participants in a Biobank study. We started this process knowing that people who live in rural areas are not well represented in Biobank studies and causes for this are not well researched. For this reason, would you be willing to take a few minutes to answer some optional questions about your decision making process for whether or not you enrolled in the Biobank? The information you provide could help future researchers develop better strategies for enrolling more representative groups in Biobank studies.

**INTERVIEW QUESTIONS:**

**1.** Before our request to join our Biobank study, had you ever heard of a Biobank?

- - Had you ever donated samples to a Biobank before? (please describe)
  - What were your initial thoughts on what a Biobank does/is?

1. What were your reasons for deciding to join the optional Biobank study?
2. [***FOR THOSE WHO DONATED BOTH A STOOL AND CHEEK SAMPLE***] Why did you decide to donate both a stool and cheek swab sample?
3. [***FOR THOSE WHO DONATED ONLY ONE SAMPLE***] Why did you decide to donate only a stool/cheek swab sample?
4. [***FOR THOSE WHO RETURNED THEIR SAMPLE(S)***] It looks like you returned your baseline samples to the Biobank. What was your experience with collecting and returning your samples to the Biobank?
   - What was helpful or challenging?
5. [***FOR THOSE WHO DID NOT RETURN THEIR SAMPLE(S)***] It looks like you did not return your baseline samples to the Biobank. Why were you unable to return you sample(s) to the Biobank?
   - Did you change your mind about participating in the Biobank?
   - Did you experience barriers in collecting your sample?
6. Is there more information that could have helped you in the sample collection process? (e.g., more instructions, more details about the process, etc.)
7. Would you be interested in more opportunities to donate to Biobank studies?

- Why or why not?

1. How is the best way to get information to you and people like you who are interested in Biobank research?
2. What could we tell others like yourself to encourage participation in Biobank research?

**Biobank 6-month Follow-up Summative Interview for Participants who Declined Enrollment**

Recruitment PID__________ Enrollment PID_______________ Date: _________

**INTRO AND PURPOSE STATEMENT:**

As part of the consent process for joining this study, you learned about an Additional optional study you could participate in with the purpose of creating a Biobank for future research. A Biobank is like a regular bank, but instead of depositing money for future spending, we are depositing biological samples for future research. The general idea of this Biobank was to collect gut microbiome and genetic samples through stool sample and cheek swab sample donations. This would potentially allow researchers to study the effects of sugary drinks on gut health and genetic functioning and would allow researchers to follow iSIPsmarter participants to examine how lifestyle changes may impact overall health.

This is our lab's first experience with enrolling participants in a Biobank study. We started this process knowing that people who live in rural areas are not well represented in Biobank studies and causes for this are not well researched. For this reason, would you be willing to take a few minutes to answer some optional questions about your decision making process for whether or not you enrolled in the Biobank? The information you provide could help future researchers develop better strategies for enrolling more representative groups in Biobank studies.

**INTERVIEW QUESTIONS:**

**1.** Before our request to join our Biobank study, had you ever heard of a Biobank?

- - Had you ever donated samples to a Biobank before? (please describe)
  - What were your initial thoughts on what a Biobank does/is?

1. What were your reasons for deciding not to join the optional Biobank study?
   - What were your barriers and concerns?
2. What might help you overcome your hesitancy to donate biological samples to a Biobank?
3. What is the best way for you to learn more about Biobank research?
4. How could we encourage others like yourself to participate in Biobank research?
